# Histological Evaluation of Internal Dental Resorption: An Analysis of a Cohort of 50 Cases

**DOI:** 10.1155/2024/1454079

**Published:** 2024-06-27

**Authors:** Tania Puentes-Morelos, Víctor Simancas-Escorcia, Arnulfo Tarón-Dunoyer, Carlos M. Ardila, Antonio Díaz-Caballero

**Affiliations:** ^1^ Universidad del Sinú, Cartagena, Colombia; ^2^ University of Cartagena, Cartagena, Colombia; ^3^ University of Antioquia, Medellín, Colombia

## Abstract

**Objective:**

This study aimed to perform a histological evaluation of teeth diagnosed with internal root resorption.

**Materials and Methods:**

A descriptive study involved the examination of 50 human teeth extracted due to an unfavorable prognosis for retention in the oral cavity. Teeth were preserved in 10% buffered formalin and subsequently subjected to the decalcification process. Masson–Goldner staining was applied for comprehensive histological assessment.

**Results:**

In all the 50 teeth examined, resorption gaps within the dentin tissue were identified, accompanied by the presence of reparative cells in the vicinity of these cavities. Marked structural loss and dentin fragmentation were evident, with regions exhibiting fissures and an absence of dentinal tubules.

**Conclusions:**

The histological evaluation of 50 teeth diagnosed with internal dental resorption revealed significant structural alterations, including resorption lacunae, the presence of multinucleated osteoclast-like cells, and reparative connective tissue. These findings highlight the complex and multifaceted nature of internal dental resorption. These histological insights provide a deeper understanding of the pathological processes involved in internal dental resorption and underscore the necessity for early detection and intervention to mitigate tooth loss and preserve dental health.

## 1. Introduction

Tooth resorption is an erosive process that compromises the integrity of tooth structure and can lead to eventual tooth loss [1]. It can be associated with physiological or pathological processes causing the loss of dentin, cementum, or bone [2]. Internal root resorption, a specific category of pulpal disease, is characterized by the progressive destruction of intraradicular dentin and dentinal tubules due to the activity of resorptive cells stimulated by inflammation of the dental pulp tissue [3].

The prevalence of internal dental resorption is difficult to ascertain due to insufficient identification data, both clinically and radiographically. Existing studies report varying prevalence rates. For example, Dao et al. [1] reported a 9.6% prevalence in a study of 171 patients, highlighting the variability based on the sample and methods used. Additionally, other studies suggest a lower prevalence, often under 2%, but these estimates are hampered by the retrospective nature of data collection and the reliance on incidental findings during routine radiographic examinations. The demographic distribution also indicates a higher incidence in males and in certain age groups, particularly those aged 30–50 years; although comprehensive data across diverse populations remain limited [4]. Moreover, according to certain research, the incidence of internal resorption ranges from 0.01% to 1% [5]. Thoma [6] documented internal root resorption in one out of every 1,000 teeth. Cabrini et al. [7], on the other hand, discovered internal resorption through histological testing in 8 of 28 teeth (28%), 49–320 days after calcium hydroxide pulpotomy. Another study of 33 autotransplanted maxillary canines found that 17 (55%) had internal resorption throughout a 6-year follow-up period [8].

The diagnosis of internal root resorption presents a significant challenge in clinical practice due to the absence of unified criteria and the diverse clinical presentations of the pathology. Accurate diagnosis often requires the use of advanced imaging techniques such as cone-beam computed tomography (CBCT), which provides a more precise three-dimensional visualization of the resorptive process [9, 10].

Clinically, internal root resorption manifests as a radiolucent area around the pulp cavity, typically affecting mandibular incisors and molars. Conventional radiography, offering only a two-dimensional image, is often insufficient to determine the nature and exact location of the resorption [11]. CBCT has improved the ability to diagnose and assess the extent of resorptive lesions, contributing to better treatment planning and prognosis [6, 7, 8, 9].

The etiology of internal root resorption is multifactorial and includes traumatic injury, infections, orthodontic treatment, internal bleaching, periodontal treatment, and idiopathic factors [12]. Resorptive cells, derived from monocyte–macrophage lineage, resorb mineralized dental tissues and are termed odontoclasts when they act on dentin and cementum [3, 4]. These cells are recruited to the mineralized surfaces, where they fuse and commence resorption [13].

Histologically, internal dental resorption is characterized by resorption lacunae containing multinucleated cells and surrounding reparative connective tissue. This reflects the ongoing pathological process as well as the body's attempt at repair. The complexity of these microscopic findings underscores the necessity for detailed histological analysis to understand the underlying mechanisms and variations in the pathology [1, 2, 3, 4].

Despite extensive literature in endodontics, orthodontics, and periodontics, there is a noticeable deficiency in understanding certain aspects of dental resorption. This gap is particularly evident in internal resorption, suggesting conceptual limitations in comprehending the cellular and histological mechanisms involved. A recent study by Rabinovich et al. [14] supported this understanding, highlighting the high variability in the etiology of internal dental resorption, which can cause confusion and delay in proper diagnosis.

This study aims to address this knowledge gap by providing a comprehensive histological evaluation of teeth diagnosed with internal root resorption. This approach offers a perspective compared to studies relying solely on clinical or radiographic findings. By examining the tissues, we can gain deeper insights into the cellular and structural characteristics of the resorptive process. These detailed data are highly relevant to understanding the diverse histopathological characteristics of internal resorption and can contribute significantly to future research on diagnosis, treatment planning, and potentially even prevention. Given the variability and complexity of internal dental resorption, this study aims to perform a comprehensive histological evaluation of 50 teeth diagnosed with internal root resorption.

## 2. Material and Methods

A descriptive study was undertaken, encompassing clinical, radiographical, and histological analyses of a sample comprising 50 teeth diagnosed with internal dental resorption. These teeth, characterized by an unfavorable prognosis for retention within the oral cavity, were extracted for the purpose of histological examination.

Our study adopts a classification system based on the location and nature of the resorption activity within the tooth structure. This system incorporates an inclusive perspective on potential causes (etiology) for different resorption forms. The two main categories are internal and external resorption. Internal resorption is further subdivided into three basic types based on Abbott and Lin's [15] classification: surface resorption, inflammatory resorption, and replacement resorption.

The sample teeth originated from the clinics of the School of Dentistry at the University of Cartagena, Colombia. The samples were obtained between May 2022 and June 2023. This retrospective study obtained a signed consent from all participating patients.

Diagnosed with internal dental resorption and a poor prognosis, the teeth were atraumatically extracted under local anesthesia. Subsequently, the teeth were stored in 4% formalin until they were transported to the Oral and Molecular Physiology laboratory at the University of Paris.

The selection criteria included mature teeth with closed apices, exhibiting dentinal lesions distant from the root cementum, along with tomographic evidence of the lesion and a poor prognosis suggesting extraction as the final alternative. Moreover, the entire root and at least a portion of the crown of the tooth remained intact, and no endodontic therapy had been performed.

### 2.1. Treatment of the Samples

After extractions, the teeth were immersed in 4% buffered formalin with a pH of 7.2 for 48 hr. Demineralization was conducted in an aqueous solution containing 10% Ethylenediaminetetraacetic acid (EDTA) for 4 weeks. Following this, the teeth underwent dehydration and were embedded in paraffin. Frontal sections of 6 *μ*m were prepared using a Leica RM2125 RTS microscope. Subsequently, Masson–Goldner staining was performed for the study of teeth with resorptions [16]. This technique utilizes three dyes to achieve distinct coloration: nuclei, stain blue or black; collagen fibers (hard tissue), stain green or blue; and cytoplasm and muscle fibers (soft tissue), stain red or orange.

### 2.2. Microscopic Analysis Criteria

For hard tissues (dentin), the analysis focused on the morphology and organization of collagen fibers within the dentin, evaluating features like presence and distribution of resorption lacunae, disruption or loss of the normal dentinal structure, and sclerosis or increased mineralization of dentin.

For soft tissues, cellular composition and organization within the resorption lacunae and surrounding areas were assessed, including identification of different cell types (e.g., inflammatory cells and odontoclasts), evaluation of blood vessel presence and distribution, and analysis of any connective tissue organization or fibrosis.

The teeth were then separated in the buccolingual direction, leaving half of the root canal exposed in each half. The tooth sections were deparaffinized in xylene (Leica Biosystems) and rehydrated through a series of decreasing alcohol concentrations. Subsequently, they underwent staining with Mayer's hematoxylin for 20 min and a brief immersion in 1% acetic acid for 30 s [17]. Following this, the sections were treated with an azophloxine solution (Sigma-Aldrich) for 10 min, then a solution of phosphotungstic acid-orange G (Sigma-Aldrich) for 1 min, and finally in light green dye (Sigma-Aldrich) for 2 min [18]. Afterward, the sections were dehydrated through increasing alcohol baths and xylene. Mounting was achieved using a nonaqueous mounting medium (DPX, Sigma-Aldrich), and observations were conducted using a Leica DM 500 optical microscope connected to a photographic camera.

CBCT scans were performed on teeth using a small-volume CBCT scanner (3D Accuitomo 80; J Morita Manufacturing, Kyoto, Japan) with exposure parameters of 80 kV, 3.0 mA. The brightness and contrast of all obtained pictures were increased to improve the visibility of the resorption lesions.

## 3. Results

Table 1 presents certain characteristics of the teeth under investigation. Most of the examined teeth were anterior and had been extracted due to either caries or trauma. Orthodontic, restorative, and occlusal reasons were also causes of root resorption.

**Figure 1:**
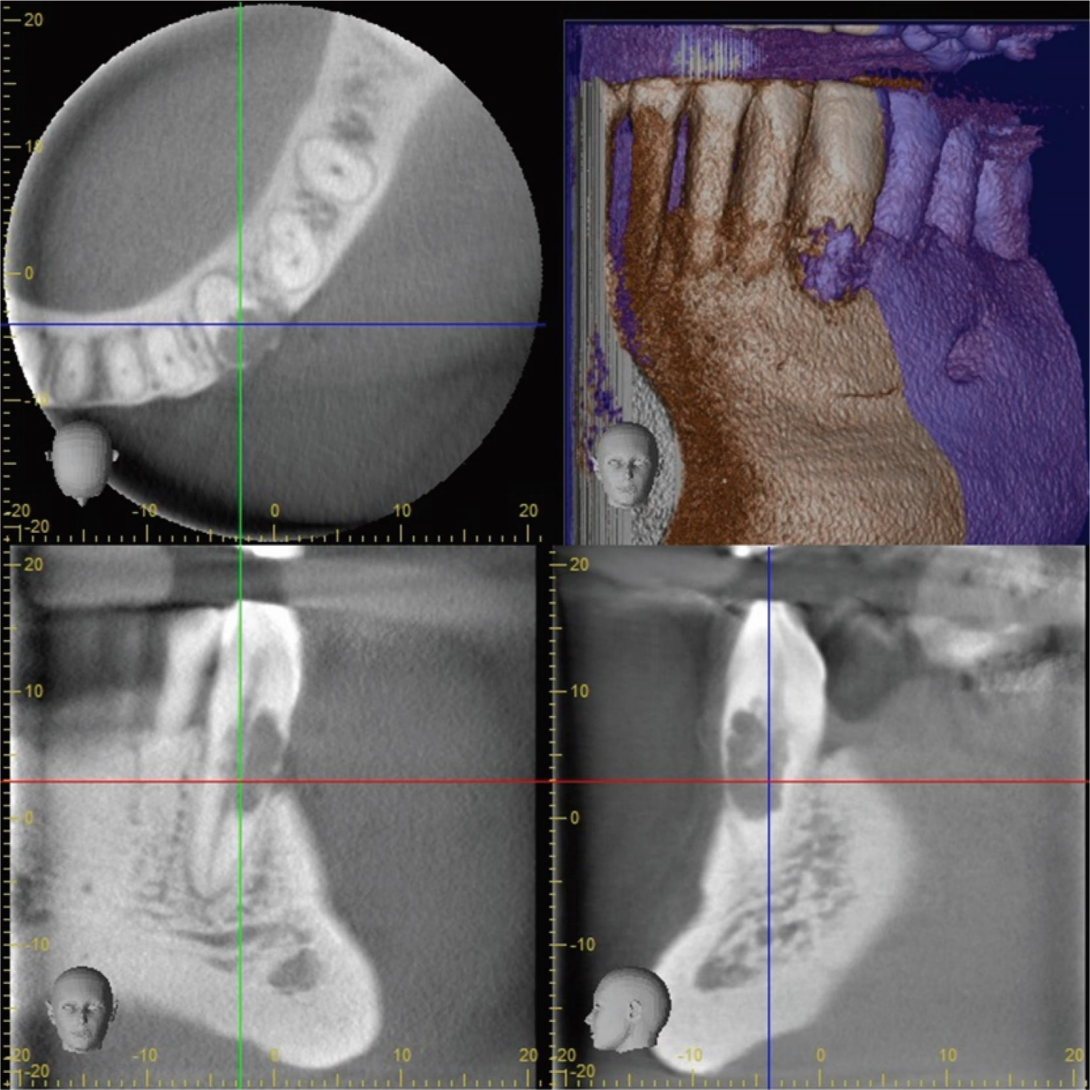
Cone-beam tomography slices enable a three-dimensional evaluation of the extent of the radiolucent area affecting the canine tooth in the cervical third toward the buccal distal region, compromising the integrity of both the root canal and the crown. The figure on the top right presents an axial plane, while the others present sagittal planes.

Figure 1 depicts a representative image of the total sample. The observations derived from tomographic sections of the examined teeth with a history of diverse issues revealed extensive levels of internal dental resorption.

**Figure 2:**
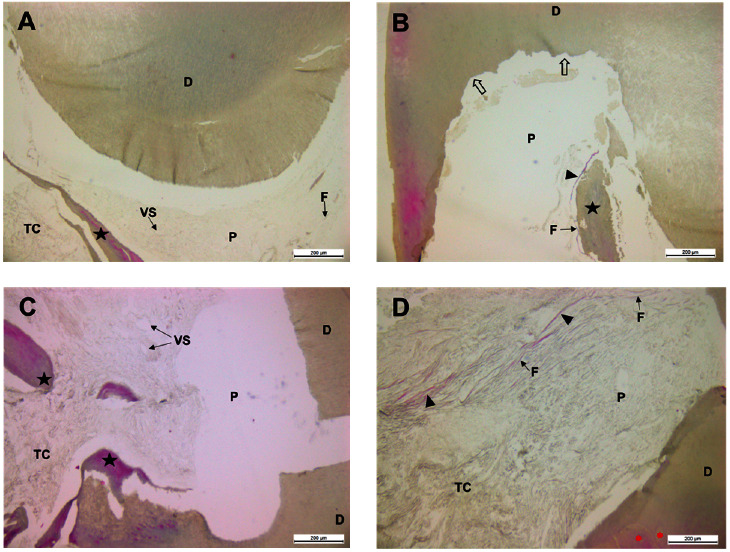
Representative microphotographs of teeth stained with Masson–Goldner staining for internal dental resorption (A–D). CT, connective tissue; D, dentin; BV, blood vessels; P, pulp; F, fibroblasts; black star, reactive or reparative tertiary dentin; black triangle, collagen fibers; red asterisks, concentric dentin regions; black bar, 200 *μ*m.

After the dental decalcification process, cuts were made in the paraffin-embedded blocks. In the sections prepared and stained with Masson–Goldner stain from the examined teeth, multiple extensive and coalescent resorption lacunae were identified, surrounded by amorphous acellular material. Structural loss and fragmentation of dentin were evident, with fissures and absence of dentinal tubules observed in some regions (Figure 2(A) and (B)). Additionally, the formation of new dentin, reactive or reparative tertiary dentin, was confirmed with a noticeable absence of odontoblasts.

Pigmented fragments reminiscent of tertiary dentin were also found within the pulp cavity. Near these elements, cellular debris was recorded (Figure 2(A), (B), and (C)). In certain areas of the dental pulp, the observed pulp tissue consisted of abundant collagen fibers located near pulp fibroblasts. The connective tissue adjacent to dentinal surfaces revealed amorphous cellular areas. In the reactive or reparative tertiary dentin near the pulp cavity, concentrically arranged dentinal regions of varying sizes and well-defined boundaries were noted (Figure 2(D)).

**Figure 3:**
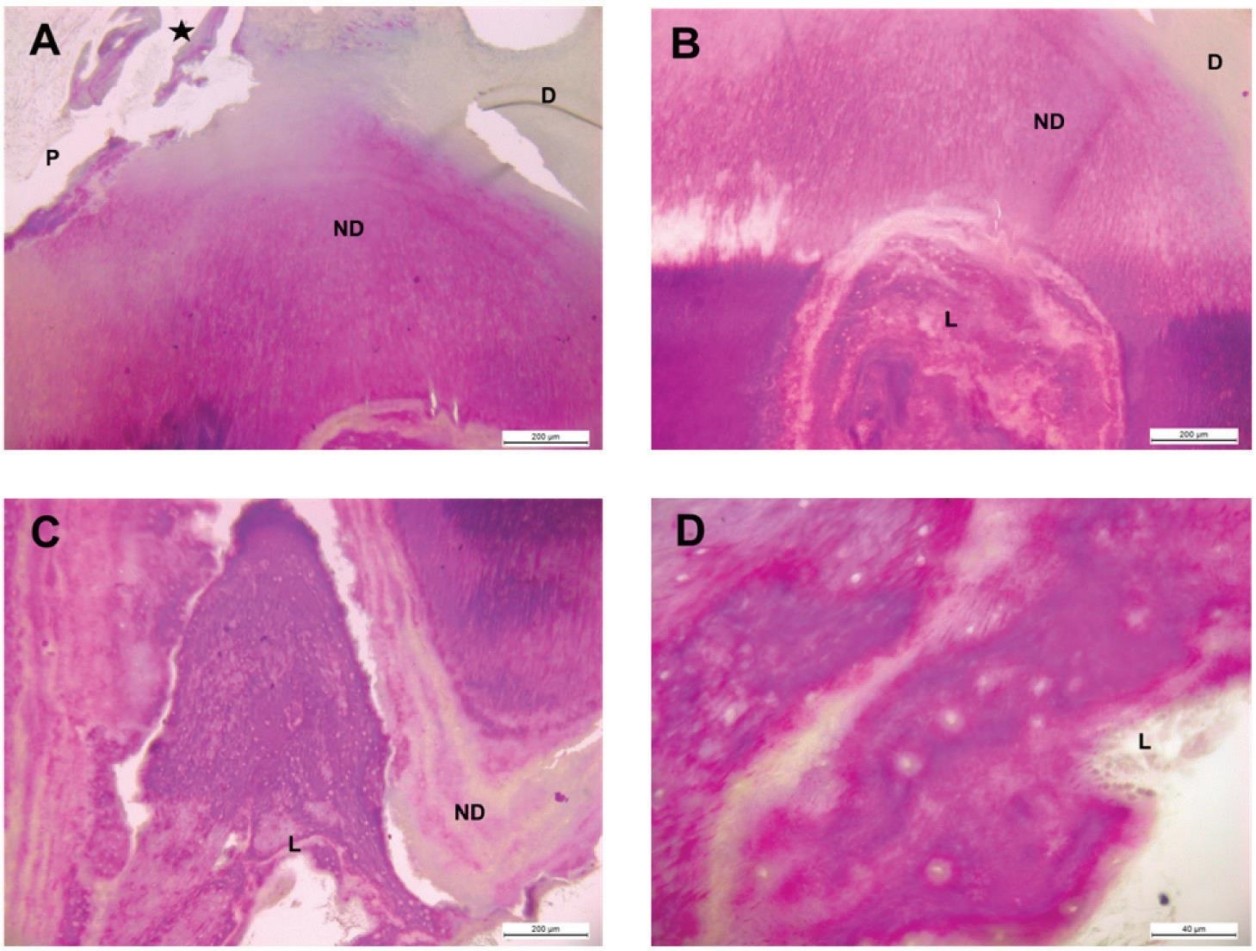
Masson–Goldner-stained pulp sections of teeth with internal dental resorption. (A) and (B) Area adjacent to dentin (D) and new dentin (ND) with resorption lacunae (L) in the pulp tissue. (C) and (D) Pulp inflammation and necrosis with resorption lacunae where odontoclasts are seen near the formation of new dentin. Black bar: A, B, and C: 200 *μ*m; D: 40 *μ*m.

Cells resembling odontoblasts were also identified in various locations within the analyzed samples. It was possible to detect and observe areas of newly formed dentin (Figures 3(A) and (B)) covering old resorption lacunae (Figures 3(B), (C), and (D)), displaying the appearance of a cellular repair attempt, as seen in inflammatory processes, indicating the simultaneous occurrence of resorption and tissue regeneration. In addition to the resorption lacunae observed in the portion of the root adjacent to the pulp cavity, odontoclasts and numerous fibroblasts were seen within these tissue lytic spaces (Figure 3(D)), along with collagen fibers adjacent to the pulp (Figure 3(A)). Furthermore, it was found that a section of the root adjacent to the periodontal ligament had several lacunae covered by reparative dentin and cementum (Figure 3(C)). Based on all this information, the frequent occurrence of pulpal inflammatory processes in cases of internal resorption is evident.

**Figure 4:**
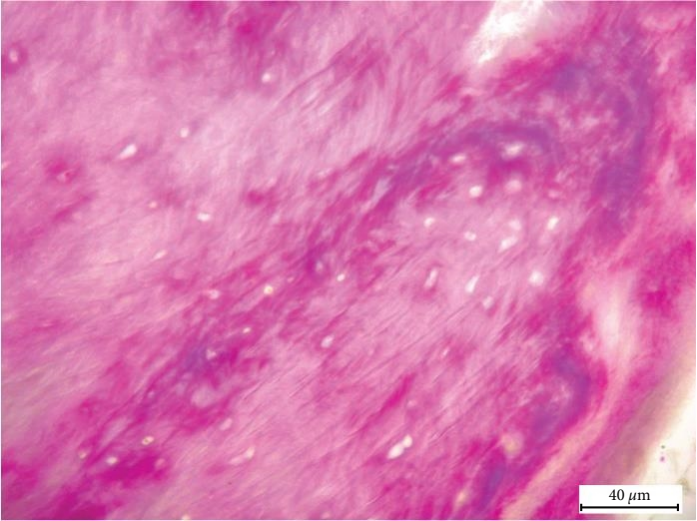
Masson–Goldner-stained pulp sections of teeth with internal dental resorption. Pulp inflammation and necrosis with resorption lacunae where odontoclasts are seen near the formation of new dentin. 40 *μ*m.

**Figure 5:**
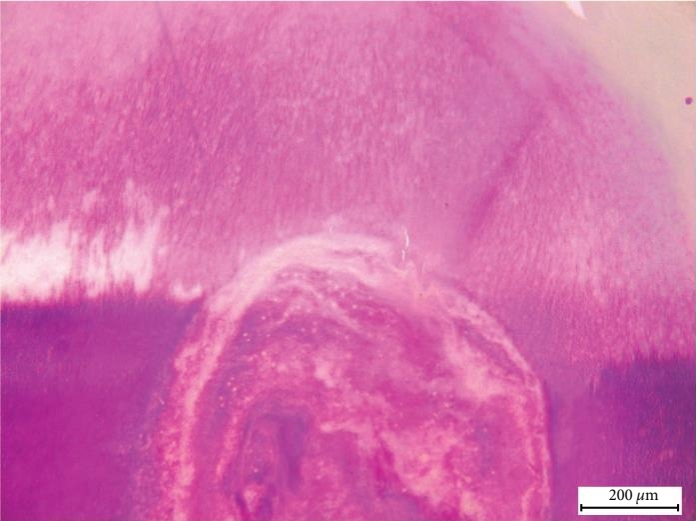
Masson–Goldner-stained pulp sections of teeth with internal dental resorption. Area adjacent to dentin and new dentin with resorption lacunae in the pulp tissue. 200 *μ*m.

**Figure 6:**
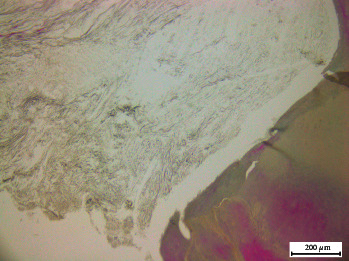
Microphotographs of teeth stained with Masson–Goldner staining for internal dental resorption. Connective tissue, dentin, and pulp are visualized. 200 *μ*m.

The Figures 4, 5, and 6 are magnified images that detail structures including pulp inflammation, necrosis, and new dentin with resorption lacunae, among others.

Table 2 summarizes some of the main histological characteristics detected in the histologically analyzed teeth. It was evident that, in addition to the chronic inflammatory reaction, a high percentage of cellular predominance consistent with an acute inflammatory process was observed in the analyzed teeth. It is worth mentioning that due to the manipulation of specimens and histological handling, it is possible that some cells or inflammatory responses may not have persisted in the evaluated sites.

## 4. Discussion

Dental pulp tissues have been implicated in the initiation or progression of internal dental resorption, as proposed by Liu et al. [19]. External resorption, on the other hand, may be triggered by trauma to the periodontal ligament. Internal tooth resorption, however, can result from injury to the residual pulp tissue. Hence, our findings regarding teeth diagnosed and treated for internal dental resorption echo previous reports, with the primary etiological agent being the inflammatory process. Notably, the resorption process may entail a histological component involving tissue replacement of mixed origin deposited within the canal, potentially leading to complete and irreparable loss of the affected tooth.

This perspective aligns with the notion posited by Patel et al. [4] and others, suggesting that internal root resorption can manifest either as primarily destructive internal inflammatory resorption or as a form accompanied by repair attempts or internal replacement resorption. This latter form is characterized by the deposition of new bone and cementum-like tissues adjacent to the resorption sites, as observed in some specimens evaluated in our research.

In a meta-analysis conducted by Souza et al. [20], the incidence of internal resorption was found to be only 1.2% in teeth subjected to intentional replantation, indicating that while this pathology may be associated with traumatic events, its occurrence is not straightforward. In contrast, the sample size of our study underscores its strength and significance. This constitutes one of the primary strengths of our study, particularly in terms of identifying internal dental resorption pathology using well-established diagnostic criteria.

While the sample size may appear limited, our study is based on a cohort of 50 teeth affected by resorption, in stark contrast to the study by Luso et al. [21], which utilized only five teeth. This discrepancy underscores the importance of our findings, providing valuable scientific insights into the significance of comprehensively investigating this complex oral pathology. Comparatively, our sample size exceeds that of the study by Gabor et al. [22], which examined a total of 30 teeth. Despite these variations, both studies unequivocally revealed the frequent occurrence of pulpal inflammatory processes in cases of internal resorption.

Dental resorption, characterized by the progressive loss of dental hard tissue, can manifest within the root or externally. Diagnosis of this condition can be challenging, and its management poses a significant clinical challenge. However, understanding the underlying pathology is imperative for elucidating the etiology and optimal management strategies. In our study, most cases originated from inflammation due to advanced dental caries or trauma, although this aspect was not the primary focus of our research. Out of the 50 teeth examined, over 50% were attributed to direct trauma or orthodontic treatment, leading to the occurrence of the internal resorptions under investigation.

While further research is warranted to delve deeper into the cellular and histological phenomena associated with internal dental resorption, including complementary immunohistochemical studies for the detection of cellular inflammation markers facilitating early detection of this pathology, histological evaluation of teeth with internal dental resorption presents inherent complexity and variable histological conditions. Various authors, such as Santos et al. [23], emphasized the indispensability of analyzing the immunohistochemical components of teeth with root resorption to enhance clarity regarding the basic science concepts underlying these oral pathologies.

Despite having a significant number of cases, we lack detailed descriptive and clinical information that would allow us to make comparisons. Future longitudinal studies with larger sample sizes that enable correlation of histological information with clinical data would be desirable.

## 5. Conclusions

The histological analysis of the evaluated sample with internal dental resorption revealed the presence of resorption lacunae, multinucleated cells, repairing connective tissue, and chronic inflammatory reactions. These findings provide insight into the histological characteristics associated with internal dental resorption. The variability and complexity observed underscore the diverse nature of this pathological process. Further research focusing on elucidating the underlying mechanisms driving internal dental resorption is warranted. Understanding these mechanisms is crucial for the development of tailored treatment strategies aimed at effectively managing this condition.

## Figures and Tables

**Table 1:** Characteristics of the evaluated teeth.

Teeth	*n*	%	Etiology
Anterior	25	50	Caries/trauma 15 Occlusion 5 Ortho/restorative 5
Molar	17	34	Caries/trauma 8 Occlusion 5 Ortho/restorative 4
Premolar	8	16	Caries/trauma 5 Occlusion 2 Ortho/restorative 1
Total	50	100	—

**Table 2:** Histological characteristics observed in the 50 evaluated teeth.

Histological features	Number of teeth	Percentage
Resorption lacunae	50	100
Multinucleated cells	35	70
Repairing connective tissue	30	60
Chronic inflammatory reaction	50	100
Acute inflammatory reaction	35	70
Soft granulation tissue	40	80

## Data Availability

The datasets used and/or analyzed during the present study are available from the corresponding author upon reasonable request.
